# Risk factors for severe neutropenia in pancreatic cancer patients treated with gemcitabine/nab-paclitaxel combination therapy

**DOI:** 10.1371/journal.pone.0254726

**Published:** 2021-07-14

**Authors:** Genta Ito, Kazuyoshi Kawakami, Takeshi Aoyama, Takashi Yokokawa, Masashi Nakamura, Masato Ozaka, Naoki Sasahira, Masayuki Hashiguchi, Hayato Kizaki, Toshihiro Hama, Satoko Hori

**Affiliations:** 1 Division of Drug Informatics, Keio University Faculty of Pharmacy, Tokyo, Japan; 2 Department of Pharmacy, Cancer Institute Hospital, Japanese Foundation for Cancer Research, Tokyo, Japan; 3 Department of Gastroenterology, Cancer Institute Hospital, Japanese Foundation for Cancer Research, Tokyo, Japan; Kaohsiung Medical University Chung Ho Memorial Hospital, TAIWAN

## Abstract

**Aim:**

Combination therapy with gemcitabine and nanoparticle albumin-bound paclitaxel (nab-paclitaxel), known as GnP therapy, significantly prolongs the survival of pancreatic cancer patients compared with gemcitabine monotherapy. However, it may cause severe neutropenia, requiring discontinuation of treatment. This study aimed to clarify the risk factors for Grade 3/4 neutropenia during GnP therapy.

**Methods:**

Clinical data of pancreatic cancer patients who underwent GnP therapy at the Cancer Institute Hospital of the Japanese Foundation for Cancer Research from December 2014 to December 2016 were retrospectively collected. The relationship of Grade 3/4 neutropenia onset to laboratory values and patient background factors was investigated by multivariate logistic regression analysis.

**Results:**

Clinical data of 222 patients were analyzed. Grade 3/4 neutropenia occurred in 118 patients (53.2%) in the first cycle of GnP therapy. Multivariate analysis identified low absolute neutrophil count (ANC), high total bilirubin (T-Bil), and low C-reactive protein (CRP) as risk factors for Grade 3/4 neutropenia. Age was not a risk factor. The incidence of neutropenia was 85.7% in patients with all three risk factors, but only 27.7% in patients with none of them.

**Conclusion:**

Low ANC, high T-Bil, and low CRP may be risk factors for Grade 3/4 neutropenia in patients receiving GnP therapy, even if these laboratory values are within normal reference ranges. Patients with these risk factors should be carefully monitored for adverse events.

## Introduction

Cancer chemotherapy may cause various adverse events, including nausea, hair loss, diarrhea, myelosuppression, liver damage and renal damage. Myelosuppression reduces neutrophils and increases the risk of developing a fatal infection. Therefore, neutropenia is a dose-limiting factor for many anticancer agents and may require a decrease in treatment intensity, such as dose reduction, postponement, or even discontinuation [1–3].

Risk factors for chemotherapy-induced neutropenia have been investigated in various dosing regimens and cancer types. A prospective observational study that analyzed data from 3,760 patients with solid tumor or malignant lymphoma identified high alkaline phosphatase (ALP), high total bilirubin (T-Bil), low glomerular filtration rate (GFR), low white blood cell (WBC) count, high relative dose intensity (RDI), and immunosuppressant administration as risk factors for severe neutropenia [4]. Other reported risk factors include female gender, renal disorder, liver dysfunction, low absolute neutrophil count (ANC) at the start of treatment, and bone metastasis [5–10].

Combination therapy with gemcitabine and nanoparticle albumin-bound paclitaxel (nab-paclitaxel), known as GnP therapy, significantly prolongs the survival of pancreatic cancer patients compared with conventional therapies such as gemcitabine monotherapy, and is considered as a standard treatment for pancreatic cancer chemotherapy, together with FOLFIRINOX (combination of 5-fluorouracil, leucovorin, irinotecan, and oxaliplatin) [11, 12]. On the other hand, myelosuppression is frequently observed, and a domestic phase II study of GnP therapy in Japan found Grade 3 or higher neutropenia in 70.6% of treated patients and febrile neutropenia in 5.9% [13]. Therefore, the target patients of GnP therapy are primarily reasonably strong patients in good general condition, and the therapy is generally considered unsuitable for elderly people. However, little is yet known about risk factors for severe neutropenia in GnP-treated patients. Recently, Makita et al. reported that low WBC and high alanine aminotransferase (ALT) levels may be risk factors for developing severe neutropenia in the first cycle of GnP therapy [14]. However, theirs was a retrospective observational study of a small population of 52 cases, and further analysis in a larger population is desirable.

In the present study, we aimed to identify the risk factors for severe (Grade 3/4) neutropenia using clinical data collected from a larger population of pancreatic cancer patients who received 1–6 cycles of GnP therapy.

## Methods

### Study setting and population

Three hundred and two patients with unresectable pancreatic cancer who underwent standard GnP therapy were targeted in this study. Data were collected from the electronic medical records of patients seen from December 1, 2014 to December 31, 2016 at the Cancer Institute Hospital of the Japanese Foundation for Cancer Research. All data were analyzed anonymously and informed consent was waived due to the retrospective observational design of the study. The data included patient background factors and clinical laboratory parameters, including sex, age, weight, body surface area (BSA), Eastern Cooperative Oncology Group Performance Status (ECOG PS), concomitant drug(s), complications, gemcitabine dose, nab-paclitaxel dose, WBC, red blood cell (RBC), hemoglobin (Hgb), platelet (PLT), ANC, lymphocyte (Lymph), albumin (ALB), T-Bil, direct bilirubin (D-Bil), γ-glutamyltransferase (γ-GTP), alkaline phosphatase (ALP), lactate dehydrogenase (LDH), aspartate aminotransferase (AST), alanine aminotransferase (ALT), serum glucose (S-Glu), urea nitrogen (UN), serum creatinine (S-Cre), estimated GFR (eGFR), C-reactive protein (CRP), and carcinoembryonic antigen (CEA).

Standard GnP therapy was administered as follows. Patients received a 30-min intravenous infusion of nab-paclitaxel at a dose of 125 mg/m^2^, followed by a 30-min intravenous infusion of gemcitabine at a dose of 1,000 mg/m^2^, on days 1, 8, and 15 every 4 weeks.

The clinical laboratory data on day 1, 8, 15 of each cycle of GnP therapy were collected. Regarding the data on days 8 and 15, a one-day deviation from the scheduled test date was allowed, but patients with a shift of two days or more were excluded. The definition of neutropenia used here was ANC <1,000/mm^3^, which is Grade 3 or higher in the Common Terminology Criteria for Adverse Events (CTCAE) version 5.0. This study was approved by the ethics committees of the Cancer Institute Hospital (approval No. 2019–1040) and the Keio University Faculty of Pharmacy (approval No. 190613–4).

### Outcome and predictor variables

The primary outcome of the study was onset of neutropenia of Grade 3 or higher. As explanatory variables for risk factor analysis, we selected sex, age, weight, BSA, ECOG PS, concomitant drug(s), complications, gemcitabine dose, nab-paclitaxel dose, and laboratory values (WBC, RBC, Hgb, PLT, ANC, Lymph, ALB, T-Bil, D-Bil, γ-GTP, ALP, LDH, AST, ALT, S-Glu, UN, S-Cre, eGFR, CRP) on day 1 of the first cycle (before administration). These risk factors were selected with reference to previous reports [4–10].

### Statistical analysis

Univariate analyses (Mann-Whitney U test, χ^2^ test) comparing the Grade 3/4 neutropenia group and the non-neutropenia group were performed for each candidate variable described above. Multivariate logistic regression analysis was performed by the simultaneous forced entry method for the variables with p<0.1 in the univariate analyses, as well as factors considered likely to be relevant based on the medical/pharmacological findings. Quantitative variables were analyzed by receiver operating characteristic (ROC) curve analysis to find the cutoff value that maximizes the Youden index, and then were stratified above and below the cutoff value. Qualitative variables were converted into dummy variables. A *p* value less than 0.05 was considered statistically significant, and the odds ratio (OR) and 95% confidence interval (CI) were calculated for each factor. The number of explanatory variables input to the multivariate logistic regression analysis was set to one-tenth of the number of event occurrence samples [15].

For statistical analysis, statistical software IBM SPSS Statistics 25.0 (SPSS Inc., Chicago, IL, USA) was used.

## Results

### Patient characteristics and incidence of neutropenia

**Fig 1:**
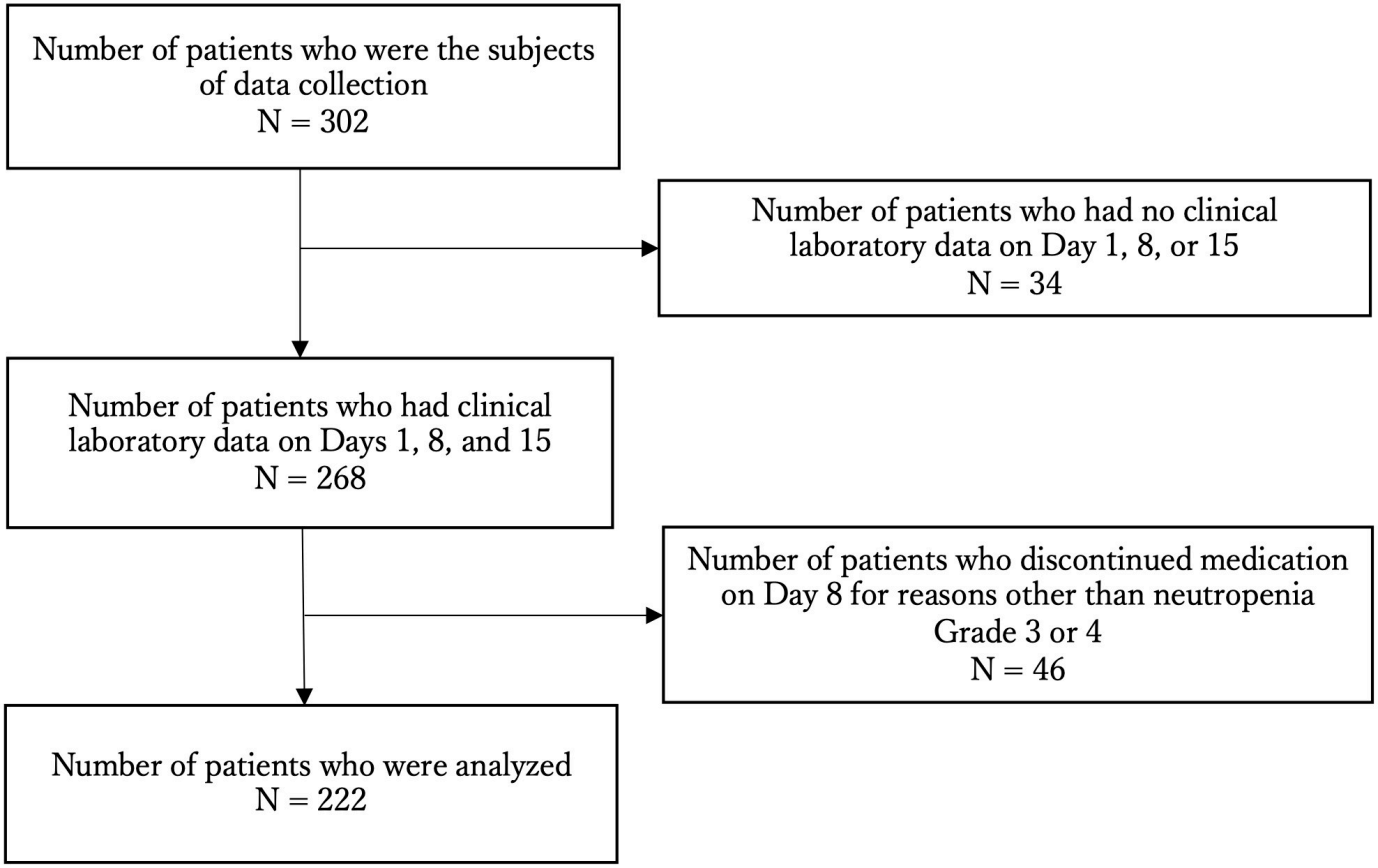
Patients included in this study.

Of the 302 patients for whom data were collected, 34 were excluded because of the absence of clinical laboratory data on Day 1, 8, or 15 of the first cycle. We also excluded 46 patients who were discontinued on Day 8 of the first cycle for reasons other than neutropenia Grade 3/4 (physical condition, peripheral neuropathy, anemia, neutropenia < Grade 3). Finally, 222 patients were analyzed in this study (Fig 1). The 222 patients analyzed in this study had a median age of 66 years (range 41–82). As summarized in Table 1, 15.3% were ≥ 75 years old, 117 were male, 79.3% were ECOG PS (0) and 20.7% were PS (1), 39.1% had liver metastasis, 30.6% had a history of chemotherapy. Of the 222 patients, 18 patients (8.1%) required starting dose reduction. The characteristics of these patients were compared with those of all patients, and they tended to include a higher proportion of patients aged > 75 years old (33.3% vs 15.3%), and those with previous chemotherapy history (55.5% vs 30.6%).

**Table 1:** Characteristics of patients at baseline.

Characteristics	Number of patients (%)
Age	
Median (yr.)	66
Range (yr.)	41–82
Distribution	188 (84.6) / 34 (15.3)
< 75 yr. / ≧75 yr.	
Gender	105 (47.2) / 117 (52.7)
Female / Male	
ECOG Performance Status	176 (79.3) / 46 (20.7)
0 / 1	
Pancreatic tumor location	
Head	106 (47.8)
Body	77 (34.7)
Tail	23 (10.3)
Others	16 (7.2)
Surgery	60 (27.0) / 162 (73.0)
Yes / No	
Disease extent	
Metastatic	85 (38.2)
Locally advanced	77 (34.6)
Postoperative recurrence	60 (27.0)
Site of metastatic disease	87 (39.1) / 29 (13.0) / 17 (7.6) / 12 (5.4)
Liver / Lung / Peritoneum /Others	
Previous chemotherapy	68 (30.6) / 154 (69.4)
Yes / No	
Regimen	40 (18.0) / 19 (8.5) / 7 (3.2) / 11 (5.0)
S-1 / FLX / GEM / others	
GEM/nab-PTX dose reduction	18 (8.1) / 204 (91.9)
Yes / No**	

Total number of patients N = 222.

ECOG; Eastern Cooperative Oncology Group, FLX: mFOLFIRINOX, GEM; gemcitabine.

**Yes: Patient with GEM/nab-PTX dose reduction, No: Patient without GEM/nab-PTX dose reduction.

**Fig 2:**
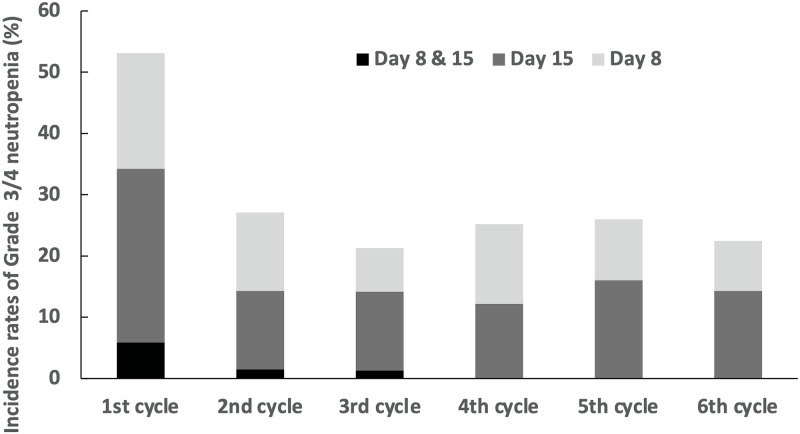
Incidence of neutropenia Grade 3/4 in each treatment cycle from 1 to 6. Day 8&15 (Black): Neutropenia developed on Day 8 and the drug was discontinued, but Grade 3 or higher neutropenia continued until Day 15. Day 15 (Dark gray): Neutropenia was absent on Day 8 but developed on Day 15 and the drug was discontinued. Day 8 (Light gray): Neutropenia developed on Day 8 and the drug was discontinued, but it was reinstated on Day 15.

The incidence of Grade 3/4 neutropenia in patients who started each cycle (1 to 6 cycles) of standard GnP therapy is shown in Fig 2. Of the 222 patients, 118 developed Grade 3/4 neutropenia; 96 (81.4%) and 22 (18.6%) of 118 patients had Grade 3 and 4 neutropenia, respectively. Of the patients with Grade 3 neutropenia, the numbers of patients who had fever were as follows: 1 patient on day 8, 1 patient on day 15, and 3 patients on both days 8 and 15. Of the patients with Grade 4 neutropenia, the numbers of patients who had fever, that is febrile neutropenia, were as follows: 1 patient on day 15, and 3 patients on both days 8 and 15. GnP therapy was discontinued in all patients who developed G3/G4 neutropenia with or without fever. The incidences of Grade 3/4 neutropenia in the first cycle and in the second or a subsequent cycle of GnP therapy were 53.2% and 25%, respectively. It should be noted that patients who developed Grade 3/4 neutropenia and discontinued the drug in the first cycle were sometimes subsequently given a reduced dose regimen, or a schedule change to biweekly administration, or were switched to a different therapy.

The incidence of neutropenia in the 18 patients who received a reduced starting dose was 56% (9 patients with Grade 3, 1 patient with Grade 4 neutropenia), which was almost the same as that of all subjects.

### Univariate analysis

The results of univariate analysis using the Mann-Whitney U test for continuous variables and the Pearson’s chi-square test for qualitative variables are shown in Table 2. Liver metastases, WBC, PLT, ANC, T-Bil, γ-GTP, ALP, LDH, CRP and CEA showed values of p < 0.1. CRP values in the severe neutropenia and non-neutropenia groups were 0.43 ± 1.10 mg/dL and 0.89 ± 1.57 mg/dL, respectively.

**Table 2:** Univariate analysis of risk factors of neutropenia Grade 3/4 in patients receiving GnP therapy.

Parameter, unit	Grade 0–2 Median (1^st^ quartile– 3^rd^ quartile)	Grade 3–4 Median (1^st^ quartile– 3^rd^ quartile)	*p-value*
Gender (Male / Female) **	52/52	65/53	0.449
Age, yr.*	65.5 (58.0–72.0)	66.5 (60.2–71.8)	0.850
Previous mFFX treatment history (Positive / Negative) **	12/92	7/111	0.136
PS (0 / 1) **	79/25	97/21	0.252
Liver metastasis (Positive / Negative) **	50/54	37/81	0.011
Body surface area, m^2^*	1.54 (1.42–1.69)	1.58 (1.45–1.65)	0.618
nab-PTX dose, mg*	188.75 (173.44–211.25)	195.00 (176.25–206.25)	0.582
GEM dose, mg*	1520.0 (1390.0–1682.5)	1560.0 (1400.0–1672.5)	0.675
GEM/nab-PTX dose reduction, Yes / No **	8/96	10/108	0.831
WBC, ×10^3^/μL*	6.0 (5.1–7.9)	5.1 (4.0–6.1)	0.000
RBC, ×10^3^/μL*	4.03 (3.57–4.30)	3.88 (3.60–4.24)	0.363
Hgb, g/dL*	12.4 (11.2–13.3)	12.3 (11.4–13.4)	0.925
PLT, ×10^3^/μL*	235 (181–280)	210 (165–246)	0.013
ANC, ×10^3^/μL*	3.90 (3.23–5.46)	3.00 (2.28–3.83)	0.000
Lymph, ×10^3^/μL*	1.35 (1.06–1.80)	1.33 (1.00–1.64)	0.641
ALB, g/dL*	3.9 (3.5–4.1)	4.0 (3.6–4.2)	0.135
T-Bil, mg/dL*	0.5 (0.4–0.7)	0.6 (0.4–0.8)	0.047
D-Bil, mg/dL*	0.2 (0.1–0.4)	0.2 (0.1–0.3)	0.480
γ-GTP, U/L*	74 (31–205)	42 (24–111)	0.006
ALP, U/L*	392 (245–548)	284 (210–428)	0.004
LDH, U/L*	191 (166–228)	180 (161–202)	0.034
AST, U/L*	26(19–35)	24 (18–35)	0.753
ALT, U/L*	24 (15–43)	23 (15–36)	0.449
S-Glu, mg/dL*	124 (106–151)	114 (103–145)	0.315
UN, mg/dL*	13 (10–16)	13 (10–15)	0.846
S-Cre, mg/dL*	0.61 (0.54–0.77)	0.64 (0.53–0.80)	0.626
eGFR, mL/min/1.73 m^2^ *	80.8 (72.4–98.4)	81.9 (70.3–94.6)	0.757
CRP, mg/dL*	0.28 (0.10–0.86)	0.10 (0.04–0.33)	0.000
CEA, ng/mL*	5.1 (2.7–22.2)	4.0 (2.4–7.7)	0.088
CA19-9, U/mL*	766.0 (78.4–18849.0)	588.4 (132.8–2847.6)	0.619

FLX: mFOLFIRINOX, GEM; gemcitabine.

*: Mann-Whitney U test,

**: Pearson’s chi-square test.

### Multivariate logistic analysis

Multiple logistic regression analysis was conducted by the simultaneous forced entry method for the items selected according to the following criteria and for the following reasons (Table 3).

- Patient background characteristics (sex, age) and factors with p < 0.1 in univariate analysis and < 10% missing values (liver metastasis, WBC, PLT, ANC, T-Bil, γ-GTP, ALP, CRP).
- For WBC/ANC (r = 0.9) and γ-GTP/ALP (r = 0.6), which showed high Spearman’s correlation coefficients, only ANC and ALP were used. For WBC/ANC, ANC was used because the objective variable was neutropenia ≥ Grade 3. For γ-GTP/ALP, ALP was used because of a low rate of missing values.
- ALT was included based on previous findings [14].
- BSA was included because GEM and nab-PTX doses were calculated based on BSA.

**Table 3:** Logistic regression analysis of the risk factors of neutropenia Grade 3/4 in patients receiving GnP therapy.

	Objective	Reference	Odds ratio	95% CI		*p-*value
Gender	Female	Male	1.177	0.503	2.754	0.707
Age	68 yr ≦	< 68 yr	1.183	0.608	2.305	0.621
Body surface area	1.64 m^2^ ≦	< 1.64 m^2^	2.454	0.995	6.054	0.051
Liver metastasis	Positive	Negative	0.874	0.439	1.737	0.700
PLT	< 229 ×10^3^ /μL	229 ×10^3^ /μL ≦	1.757	0.932	3.315	0.082
ANC	< 3.03×10^3^/μL	3.03×10^3^/μL ≦	4.806	2.416	9.558	0.000
ALP	< 346 U/L	346 U/L ≦	1.49	0.685	3.243	0.315
ALT	< 41 U/L	41 U/L ≦	1.349	0.587	3.1	0.481
CRP	< 0.13 mg/dL	0.13 mg/dL ≦	2.607	1.331	5.106	0.005
T-Bil	0.6 mg/dL ≦	< 0.6 mg/dL	1.964	1.04	3.708	0.037

The number of explanatory variables in the multiple logistic regression analysis was selected to be 10 which is less than one-tenth of the number of event occurrence samples (neutropenia 118 and non-neutropenia 104 patients) [15]. As factors with p < 0.05, we identified ANC < 3.03×10^3^ /μL (OR: 4.806, 95% CI: 2.416–9.558, p = 0.000), T-Bil ≥ 0.6 mg/dL (OR: 1.964, 95% CI: 1.040–3.708, p = 0.037), and CRP < 0.13 mg/dL (OR: 2.607, 95% CI: 1.331–5.106, p = 0.005).

### Comparison of incidence rates of neutropenia according to the number of risk factors

**Fig 3:**
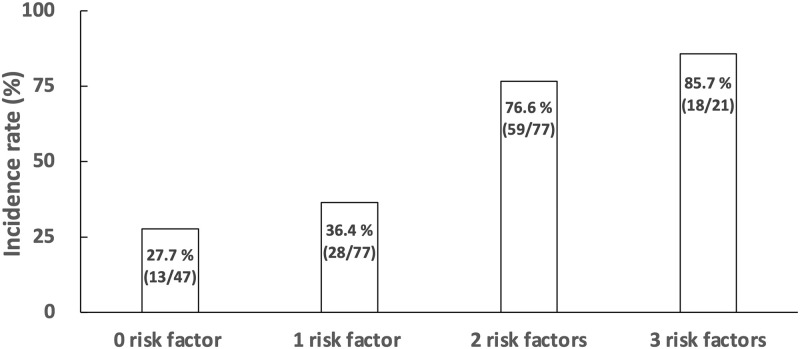
Comparison of incidence rates of neutropenia Grade 3/4 by number of risk factors. Patients were divided according to their number of risk factors (ANC < 3.03 × 10^3^ /μL, T-Bil ≥ 0.6 mg/dL, and CRP < 0.13 mg/dL), and the incidence of neutropenia Grade 3/4 was calculated in each group.

The incidence of Grade 3/4 neutropenia in the first cycle of GnP therapy was 53.2%. The incidence was 85.7% in patients with all three risk factors (ANC < 3.03×10^3^ /μL, T-Bil ≥ 0.6 mg/dL, CRP < 0.13 mg/dL) and 27.7% in patients without any of these risk factors (Fig 3).

## Discussion

In the present study, we clarified the incidence and risk factors of severe neutropenia (> Grade 3) due to GnP therapy with or without prior treatment in Japanese patients with unresectable pancreatic cancer by the largest retrospective observational study so far.

### Incidence rate of severe neutropenia

The incidence of neutropenia ≥ Grade 3 in the first cycle of GnP therapy in patients with pancreatic cancer was 53.2% in this study, whereas Braiteh et al [16]. reported an incidence of 28% in patients with metastatic pancreatic cancer and an incidence of 4% in patients with a previous history of chemotherapy. However, Makita et al [14]. reported an incidence of 61.5% (32/52) in unresectable pancreatic cancer patients in Japan, and Yano et al [17]. found that the incidence of neutropenia due to docetaxel in clinical trials conducted in Asia was higher than in non-Asians. GnP therapy includes paclitaxel, which is a taxane-based anticancer drug similar to docetaxel. Therefore, the difference in the incidence of severe neutropenia between our study and that of Braiteh et al. [16] may reflect racial differences. This view is supported by the fact that the subjects in our study were similar to those of Makita et al. [14] in terms of country of origin, disease extent, and previous chemotherapy history, and the incidence of severe neutropenia in our study was close to that found in their study.

### Risk factors for severe neutropenia

We identified ANC < 3.03 × 10^3^ /μL, T-Bil ≥ 0.6 mg/dL, CRP < 0.13 mg/dL as risk factors for Grade 3/4 neutropenia in standard GnP therapy (CTCAE ver5.0). The reference ranges are ANC ≧ 2.0 × 10^3^ /μL, T-Bil 0.4–1.5 mg/dL, and CRP 0.00–0.14 mg/dL [18], so the criterion values for increased risk fall within the reference ranges. Although T-Bil and CRP have not been previously reported as risk factors for Grade 3/4 neutropenia in GnP therapy, our results suggest that close monitoring of adverse events is important even in patients whose clinical laboratory values are within the reference ranges.

Low ANC level has been proposed to be a risk factor of neutropenia in cancer chemotherapy with various agents [19], not only GnP, so it could be a risk factor for neutropenia regardless of the regimen.

As regards T-Bil, Joerger et al. reported that this parameter is a good predictor of paclitaxel excretion and paclitaxel-induced myelosuppression [20]. Thus, it seems likely that patients with high T-Bil levels in the present study would have had an increased blood concentration of paclitaxel due to decreased clearance, and this could have caused neutropenia.

CRP in neutropenia Grade 0–2 was higher than that in Grade 3/4 (Grade 0–2: 0.28 mg/dL, Grade 3/4: 0.10 mg/dL). Thus, low CRP level might be a risk factor, because ANC would remain high in patients with high CRP level due to increased inflammatory response, and ANC tended to be low in patients with low CRP level. On the other hand, Razzaghdoust et al. reported that high CRP (≥ 0.6 mg/dL) is a risk factor for severe neutropenia or febrile neutropenia in patients with breast cancer and gastrointestinal cancer [21]. In their study, the CRP (mean ± SE) was 2.46 ± 0.06 mg/dL in patients with severe neutropenia or febrile neutropenia and 1.29 ± 0.17 mg/dL in patients without severe neutropenia or febrile neutropenia. In the present study, the CRP values in the severe neutropenia and non-neutropenia groups were 0.43 ± 1.10 mg/dL and 0.89 ± 1.57 mg/dL, respectively. Patients with PS = 2, who might have been predicted to have higher CRP values were not included in this analysis. A possible explanation for the apparent discrepancy is that pancreatic cancer patients with high CRP may not have been selected for GnP therapy, i.e., there could be selection bias in our study.

Since the incidence of pancreatic cancer increases with age, it is important to assess the suitability of chemotherapy regimens for the elderly. At present, GnP therapy generally tends not to be offered to elderly people (≥ 75 years) because of safety concerns. However, we did not find that age is a risk factor for neutropenia, although this study included a small proportion of elderly people (34 patients, 15.3%), who had either PS 0 (26 patients) or PS 1 (8 patients). Therefore, GnP therapy may be an option in elderly people (≥ 75 years) with good PS.

### Strengths and limitations

This study is the largest retrospective observational study so far conducted to evaluate risk factors for severe neutropenia in patients treated with GnP therapy. This study design inevitably has various potential limitations, including selection bias, variations of blood sampling date/time, parameters evaluated, inclusion of patients whose dose regimen had to be altered, differences in patients’ characteristics, and so on. These limitations should be noted when interpreting the results of this study.

## Conclusions

Our findings indicate that low ANC, high T-Bil, and low CRP are risk factors for severe neutropenia in patients receiving GnP therapy, even if the clinical laboratory test values lie within the normal reference ranges. The incidence of neutropenia was 85.7% in patients with all three risk factors, but only 27.7% in patients with none of them. Thus, careful monitoring of adverse events is needed in patients with these risk factors who are receiving GnP.
